# Maternal HIV retesting during antenatal care in selected health facilities in Mayuge district, Uganda: A cross-sectional study

**DOI:** 10.1371/journal.pgph.0004173

**Published:** 2025-01-15

**Authors:** Farouq Kayiwa, Elizabeth Nabiwemba, Nelson Bunani, Rawlance Ndejjo

**Affiliations:** 1 Department of Epidemiology and Biostatistics, School of Public Health, College of Health Sciences, Makerere University, Kampala, Uganda; 2 Department of Community Health and Behavioural Sciences, School of Public Health, College of Health Sciences, Makerere University, Kampala, Uganda; 3 Department of Health Policy Planning and Management, School of Public Health, College of Health Sciences, Makerere University, Kampala, Uganda; 4 Department of Disease Control and Environmental Health, School of Public Health, College of Health Sciences, Makerere University, Kampala, Uganda; Federal University, Oye-Ekiti, Nigeria & Wits University, NIGERIA

## Abstract

Vertical transmission of HIV continues to be a key contributor to pediatric HIV infections globally. Routine HIV testing at each antenatal care (ANC) visit can reduce the likelihood of such infections. However, a sub-optimal number of women are re-tested for HIV on their subsequent ANC visits. This study determined the proportion of pregnant women who were retested during ANC and the associated factors in selected health facilities in Mayuge district, Uganda. This was a cross-sectional study that utilized quantitative data collection techniques among 344 randomly selected women recorded as HIV-negative at their first ANC visit in 2022 from records at selected health facilities in Mayuge district. A semi-structured questionnaire designed in Kobo Collect software was utilized to collect the data. The data were collected through in-person interviews or by phone. Descriptive data were reported for the sample, and multivariable modified Poisson regression models were fitted to estimate prevalence ratios for factors associated with HIV re-testing. Out of 344 women attending ANC, 18.6% (64/344) had been retested for HIV. The factors associated with HIV retesting included attending ANC in the second trimester (APR = 0.62; 95%CI:0.40–0.98), attending at least four ANC visits (APR = 2.24; 95% CI:1.22–4.09), having a planned pregnancy (APR = 1.96; 95%CI: 1.14–3.37), being satisfied with the quality of health services provided (APR = 1.79; 95%CI: 1.05–3.04), and having easily accessible HIV testing resources at the ANC clinic (APR = 2.04; 95%CI: 1.08–3.85). HIV retesting during ANC in Mayuge district was low and mostly influenced by ANC-related factors at the individual and systems levels. These factors ought to be addressed to improve the uptake of maternal HIV retesting.

## Introduction

Human immunodeficiency virus (HIV) continues to be a significant public health problem affecting the population globally. In 2022, an estimated 39.0 million people were living with the virus, of which 1.5million were children aged 0–14 years [1]. Forty-two percent and 21% acquired HIV vertically during breastfeeding, and due to mothers seroconverting during pregnancy or breastfeeding [2]. Although there has been use of effective HIV preventive interventions through the prevention of vertical transmission, pediatric HIV infections remain high, especially in sub-Saharan Africa (SSA), where almost 90% of the children living with HIV live [3].

Early diagnosis and effective treatment of maternal HIV infection is critical to reduce vertical transmission [4]. Without proper early detection and preventive measures, 15 to 45% of the HIV positive pregnant women can transmit the virus to their children during pregnancy, delivery and/or breastfeeding [5,6]. To avert the infections, it is crucial to integrate HIV testing in antenatal care (ANC) and ensure universal access to antiretroviral therapy (ART) for all pregnant women who test HIV positive [7]. Whereas the coverage of HIV testing at the first ANC visit is usually high through opt-out testing, retesting coverage remains low. Newly acquired HIV infections during pregnancy may go undetected as some pregnant women are not tested for HIV in subsequent ANC visits and are unaware of their HIV status by the time of delivery, which exposes the unborn babies to the risk of infection [8].

Uganda, like many other parts of SSA, is still challenged by the HIV epidemic, with an adult prevalence of 5.8%, higher among women (7.2%) compared to men (4.3%) [9]. It has been estimated that about 5,500 new childhood HIV infections in Uganda in 2021 were due to vertical transmission of HIV [10]. Routine testing of HIV at each ANC visit can avert such infections [7]. Moreover, the Uganda national guidelines for HIV testing recommend that all women attending ANC are tested for HIV, and those who are negative are retested at all subsequent ANC visits to timely identify those who may be infected for enrolment into care [11]. Despite this, a country-wide study in Uganda that used routine health facility data from the District Information System (DHIS2) found that only 6.9% of pregnant women knew their HIV status at the first ANC visit and many women did not return for subsequent ANC visits and were therefore not retested [12].

Previous studies have reported factors associated with HIV testing among pregnant women during ANC to include residence, wealth index, distance to the health facility, education level, marital status, HIV knowledge, risky sexual activity, multiple sexual partnerships, and awareness about vertical transmission of HIV [13–15]. However, these studies did not explore retesting for HIV.

There is limited information regarding the prevalence and factors associated with HIV retesting during ANC in Uganda which threatens the gains so far achieved in the prevention of vertical transmission of HIV and achieving zero HIV infections by 2030 [16]. In this study, we aimed to determine the proportion of women who received HIV retesting, and the factors associated with maternal HIV retesting during ANC in selected health facilities in Mayuge district Uganda.

## Materials and methods

### Study design and setting

This was a cross-sectional study conducted among women seeking ANC services in selected health facilities in Mayuge district in east central Uganda. The district has a total of 50 health facilities that offer ANC services. These include 34 Health centre IIs (HCIIs), 2 private clinics, 10 HCIIIs, 3 HC IVs, and 1 private not-for-profit hospitalMayuge district was selected for this study because of a high HIV prevalence of 3.4% [17], yet data on HIV retesting during ANC was limited. Moreover, data from DHIS2 for Mayuge district indicates that, less than 50% of all pregnant women who were tested for HIV in the first trimester were retested on their subsequent ANC visits across all the health facilities in the district in 2023.For our study, we purposively selected three health facilities that had a high number of pregnant mothers seen at the ANC clinic. These health facilities included Malongo HCIII, Mayuge HCIV, and Buluba Hospital.

### Sampling and sample size

Using the Kish Leslie formula for computing sample size for cross-sectional studies, a sample size of 344 respondents was computed [18]. We considered a proportion of 32% for HIV retesting among women attending ANC, based on data from Penumetsa, Neary [19]. We used a 95% confidence level and a 5% margin of error to determine the required sample size. In the selected health facilities, the ANC clinic In-charges were requested for the ANC registers for pregnant women who attended their first ANC in the last quarter of 2022 (October to December). Only pregnant women who had had a negative HIV test at the initial test were considered for the sampling frame. In the fourth quarter of 2022, Malongo HCIII registered 2061 HIV-negative women, Mayuge HCIV registered 1885, and Buluba Hospital registered 514 HIV-negative women who attended ANC. Proportionate to size sampling technique was utilized to select 159 participants from Malongo HCIII, 145 participants from Mayuge HCIV, and 40 participants from Buluba Hospital. In each of the health facilities, systematic random sampling was utilized to select the participants for interviews. A sampling interval of 13 was determined considering the total number of eligible participants at each site following a random starting point.

### Study variables

The dependent variable was HIV retesting among women attending ANC in the selected health facilities in Mayuge district. HIV retesting was defined as a woman with an initial HIV-negative result in the ANC register who had tested again at least 12 weeks after the initial test on a subsequent ANC visit as per the Uganda National guidelines for HIV testing during ANC. This was measured as a binary variable with a woman who retested being coded as 1 = Yes, and those who did not retest being coded as 0 = N0.

Independent variables were based on Andersen model of health care utilization [20,21], and classified into individual and health system-related factors. The individual factors included maternal age, marital status, source of income, occupation, education level, parity, number of ANC visits, timing of ANC, knowledge of vertical transmission of HIV, and perceived social support. Health system-related factors included waiting time, perceived quality of ANC services, healthcare provider attitudes, client satisfaction, availability of HIV testing resources at the ANC clinic, and distance to the healthcare facility. Timing of ANC was measured as the gestation age of the pregnancy at first ANC, which was categorized as 1 = first trimester, 2 = second trimester, and 3 = third trimester. Additionally, the participant’s knowledge was assessed using a set of six questions about the mode of transmission of HIV and its prevention, whether the respondent had heard about vertical transmission, how vertical transmission occurs and its prevention adapted from previous studies [22,23]. Each correct response was scored ’1’, while incorrect responses were scored ’0’. The total scores for each participant were calculated by adding up their individual scores. Subsequently, the median score was used to divide the participants into two categories: those who scored at or above the median were categorized as having a high knowledge level, whereas those who scored below the median were considered to have a low knowledge level. The perceptions regarding vertical transmission of HIV were measured using 14 statements around the perception of the risk of acquiring HIV and that of their partners, worry about HIV and potential for sickness and hospitalization, and HIV testing, among others that were measured on a 5-point Likert scale [24,25] (Additional file 1). Strongly agree and agree were merged and renamed “agree”, while neutral, disagree, and strongly agree were merged and labelled as “disagree”. Then, the responses were given scores: 1 for those who agreed and a score 0 for those who disagreed. The scores were added up, and the average was computed. A score above average was considered to have a positive perception, and those below average were considered to have a negative perception.

### Data collection

We included all pregnant women who attended their first ANC in the last quarter (October–December) of 2022 and tested negative at their first HIV test. These women were expected to have had their second ANC visit and another HIV test by the time of this study according to the guidelines. The women were recruited for interviews between 16/05/2023 and 06/06/2023. Those who were too ill to respond to the interview questions were excluded. From the register, the contacts of mothers who attended ANC were extracted, and those who met the inclusion criteria were contacted by phone and requested for an interview. The interviews were conducted either physically for those who came to the health facility or on the phone for those who did not come during the data collection period. These interviews were conducted in Lusoga (the local language spoken in the area) by a team of research assistants with a minimum of a bachelor’s degree. The research assistants underwent one day of training to equip them with research ethics principles, background of the study, and data collection skills. The data were collected using a semi-structured questionnaire (Additional file 1) designed in Kobo collect, which was administered to the respondents through face-to-face and phone interviews.

### Data management and analysis

Data were downloaded into Microsoft Excel, cleaned, and exported into STATA version 15 for analysis. Descriptive statistics such as mean, median and proportions were used to present the participants’ background characteristics.

Modified Poisson regression with robust variances was used at bivariate and multivariable analysis to identify factors associated with HIV retesting. Associations were tested at a 95% confidence interval (CI). Prevalence ratios (PR) were used instead of odds ratios because the prevalence of the outcome of interest (HIV retesting) was greater than 10%, and odds ratios tend to overestimate the strength of the association in such instances [26]. The factors which had a p-value <0.2 at bivariable analysis and those that were biologically plausible were included in the multivariable analysis model to obtain adjusted PRs. Model building was done by stepwise elimination, which involved adding the variables that qualified for multivariable level one at a time while dropping those with no significance. This was done to obtain the best model with a smaller Akaike’s information criterion (AIC) and the log-likelihood ratio closer to zero. This model was taken as the best fit. Unadjusted and adjusted prevalence ratios and their 95% confidence intervals (CIs) and p-values are reported.

## Results

### Background characteristics of the study participants

As shown in Table 1, more than half 58.7% (202/344) of the participants were aged between 20–29 years, and the mean age of mothers was 24.6 years and standard deviation of ±5.9). Just over three-quarters of respondents—76.2% (262/344) reported residing in rural areas, 59.9% (206/344) were married/cohabiting, and 41.9% (144/344) reported primary school as their highest level of education. Additionally, more than half, 53.2% (183/344) of the mothers were unemployed. Just over two-thirds, 65.7% (226/344) of the participants were multiparous, and only 43.3% (149/344) attended the first ANC in their first trimester. A majority of the study participants, 238/344 (69.2%), attended less than 4 ANC visits, and 68.95% (237/344) had unplanned pregnancies (Table 1).

**Table 1:** Socio-demographic characteristics of mothers attending ANC in the selected healthcare facilities in Mayuge district, N = 344.

Variable	Category	Frequency (n = 344)	Percentage (%)
**Age (years) (mean = 24.6, S.D ±5.9)**	Less than 20	67	19.5
	20–29	202	58.7
	30+	75	21.8
**Residence**	Rural	262	76.2
	Urban	82	23.8
**Marital status**	Single/never married	116	33.7
	Married/Cohabiting	206	59.9
	Divorced/Separated	22	6.4
**Highest education level**	No formal education	84	24.4
	Primary	144	41.9
	Secondary	116	33.7
**Main source of income**	Employed (by someone or an organization)	20	5.8
	Self-employed (Casual laborer, peasant famer, petty trade)	141	41.0
	Unemployed	183	53.2
**Parity**	Uniparous	118	34.3
	Multiparous	226	65.7
**Timing of first ANC**	First trimester	149	43.3
	Second trimester	177	51.5
	Third trimester	18	5.2
**Number of ANC visits attended**	Less than 4	238	69.2
	4 and above	106	30.8
**Planned pregnancy**	No	237	68.9
	Yes	107	31.1
**Weeks of gestation at the time of first HIV test**	First trimester	149	43.3
	Second trimester	169	49.1
	Third trimester	26	7.6
**Reported spousal support**	No	158	45.9
	Yes	186	54.1

### Maternal HIV retesting during antenatal care and associated factors

The proportion of women who had been retested for HIV during ANC in selected health facilities in Mayuge district was 18.6% (64/344). The associated factors are described below.

### Individual factors

In bivariate analysis, having a secondary level of education, performing at least 4 ANC visits, having a planned pregnancy, receiving spousal support and having a high level of knowledge about PMTCT were positively associated with HIV retesting. Furthermore, attending the first ANC during the second trimester and receiving the 1^st^ HIV test during the second trimester were negatively associated with HIV retesting. In the multivariable model, the individual factors associated with maternal HIV retesting included timing of ANC, number of ANC visits conducted and whether the pregnancy was planned. The prevalence of HIV retesting was 38% lower among respondents who attended their first ANC in the second trimester compared to those who did so in their first trimester (APR = 0.62; 95%CI:0.40–0.98). Furthermore, the respondents who attended at least 4 ANC visits, had a 2.24 times higher prevalence of HIV retesting compared to those who had attended less than 4 ANC visits (APR = 2.24; 95% CI:1.22–4.09). The prevalence of HIV retesting was 96% higher among mothers who reported that their pregnancy was planned compared to those with unplanned pregnancy (APR = 1.96; 95%CI: 1.14–3.37) (Table 2).

**Table 2:** Health system and individual factors associated with maternal HIV retesting during ANC in selected healthcare facilities in Mayuge district, Uganda.

Variable	Maternal HIV retesting		UPR [95% CI]	P-value	APR [95% CI]	P-value
	Yes (N = 64) %	No (N = 280) %				
**Age**						
Less than 20	12 (18.8)	55 (19.6)	1 (ref)		1 (ref)	
20–29	40 (62.5)	162 (57.9)	1.11 (0.62–1.98)	0.736	1.03 (0.55–1.26)	0.925
30+	12 (18.8)	63 (22.5)	0.89 (0.43–1.85)	0.762	0.85 (0.40–1.79)	0.664
**Residence**						
Rural	48 (75.0)	214 (76.4)	1 (ref)		1 (ref)	
Urban	16 (25.0)	66 (23.6)	1.07 (0.64–1.77)	0.808	0.72 (0.41–1.26)	0.249
**Highest education level**						
No formal education	8 (12.5)	76 (27.1)	1 (ref)		1 (ref)	
Primary	27 (42.2)	117 (41.8)	1.97 (0.94–4.14)	0.074	1.29 (0.54–3.08)	0.570
Secondary	29 (25.3)	87 (31.1)	2.60 (1.26–5.46)	0.010	1.30 (0.51–3.30)	0.581
**Main source of income**						
Employed	27 (42.2)	156 (55.7)	1 (ref)		1 (ref)	
Self-employed	32 (50.0)	109 (38.9)	1.54 (0.97–2.44)	0.069	0.85 (0.51–1.42)	0.532
Unemployed	5 (7.8)	15 (5.4)	1.69 (0.73–3.91)	0.217	0.80 (0.32–2.06)	0.651
**Parity**						
Uniparous	28 (43.8)	90 (32.1)	1 (ref)		1 (ref)	
Multiparous	36 (56.3)	190 (67.9)	0.67 (0.43–1.04)	0.077	0.81 (0.48–1.36)	0.422
**Timing of first ANC care**						
First trimester	41 (64.1)	108 (38.6)	1 (ref)		1 (ref)	
Second trimester	21 (32.8)	156 (55.7)	0.43 (0.27–0.70)	0.001	0.62 (0.40–0.98)	0.042
Third trimester	2 (3.1)	16 (5.7)	0.40 (0.11–1.53)	0.183	0.74 (0.20–2.81)	0.664
**Number of ANC visits**						
<4	22 (34.4)	216 (77.1)	1 (ref)		1 (ref)	
≥4	42 (65.6)	64 (22.9)	4.29 (2.70–6.81)	<0.001	2.24 (1.22–4.09)	0.009
**Planned pregnancy**						
No	27 (42.2)	210 (75.0)	1 (ref)		1 (ref)	
Yes	37 (57.8)	70 (25.0)	3.04 (1.95–4.72)	<0.001	1.96 (1.14–3.37)	0.015
**Weeks of gestation during the 1st HIV test**						
First trimester	39 (60.9)	110 (39.3)	1 (ref)			
Second trimester	23 (35.9)	146 (52.1)	0.52 (0.22–0.83)	0.006		
Third trimester	2 (3.1)	24 (8.6)	0.29 (0.08–1.15	0.078		
**Spousal support**						
No	25 (39.1)	133 (47.5)	1 (ref)		1 (ref)	
Yes	39 (60.9)	147 (52.5)	1.33 (0.84–2.09)	0.001	0.97 (0.61–1.54)	0.907
**Knowledge of PMTCT**						
Low	18 (28.1)	133 (47.5)	1 (ref)		1 (ref)	
High	46 (71.9)	147 (52.5)	2.00 (1.21–3.30)	0.003	1.47 (0.82–2.64)	0.196
**Perceptions regarding PMTCT**						
Negative	26 (40.6)	145 (51.8)	1 (ref)		1 (ref)	
Positive	38 (59.4)	135 (48.2)	1.44 (0.92–2.27)	0.111	0.89 (0.55–1.42)	0.619
**Client satisfaction**						
Not satisfied	16 (25.0)	138 (49.3)	1 (ref)		1 (ref)	
Satisfied	48 (75.0)	142 (50.7)	2.43 (1.44–4.11)	0.001	1.79 (1.05–3.04)	0.033
**Distance to the nearest Health facility**						
<5km	33 (51.6)	90 (32.1)	1 (ref)		1 (ref)	
≥5km	31 (48.4)	190 (67.9)	0.52 (0.34–0.81)	0.004	0.81 (0.50–1.32)	0.393
**Given information on HIV testing while at the healthcare facility**						
No	13 (20.3)	97 (34.6)	1 (ref)		1 (ref)	
Yes	51 (79.7)	183 (65.4)	1.84 (1.05–3.25)	0.034	0.91 (0.49–1.71)	0.779
**Offered HIV testing at the ANC clinic during your antenatal care visits**						
No	5 (7.8)	45 (16.1)	1 (ref)		1 (ref)	
Yes	59 (92.2)	235 (83.9)	2.01 (0.85–4.76)	0.114	1.04 (0.39–2.75)	0.994
**Delayed to obtain HIV test results from the ANC clinic**						
No	41 (64.1)	114 (40.7)	1 (ref)		1 (ref)	
Yes	23 (35.9)	166 (59.3)	0.46 (0.29–0.73)	0.001	0.67 (0.39–1.13)	0.133
**HIV testing resources were available at the ANC clinic**						
No	10 (15.6)	121 (43.2)	1 (ref)		1 (ref)	
Yes	54 (84.4)	159 (56.8)	3.32 (1.75–6.30)	<0.001	2.04 (1.08–3.85)	0.029
**Received pre-test counselling before getting an HIV test**						
No	17 (26.6)	141 (50.4)	1 (ref)		1 (ref)	
Yes	47 (73.4)	139 (49.6)	2.35 (1.41–3.92)	0.001	1.11 (0.64–1.93)	0.716

UPR-Unadjusted Prevalence Ratio; APR-Adjusted Prevalence Ratio; CI-Confidence Interval

### Health system factors

In bivariate analysis, being satisfied with the ANC services provided, receiving information on HIV testing while at the healthcare facility, easy access to HIV testing resources and receiving pre-test counselling before getting an HIV test were positively associated with HIV retesting. A distance of at least 5 km to the nearest healthcare facility and reported delays in getting HIV test results from the ANC clinic were negatively associated with HIV retesting. In the multivariable model, the health system factors associated with HIV retesting were client satisfaction with ANC services and accessibility of HIV testing resources at the ANC clinic. The prevalence of maternal HIV retesting was 79% higher among mothers who were satisfied with the quality of health services provided compared to those who were not (APR = 1.79; 95%CI: 1.05–3.04). In addition, the prevalence of HIV retesting was 2.04 times among mothers who reported that HIV testing resources at the ANC clinic were easily accessible compared to their counterparts (APR = 2.04; 95%CI: 1.08–3.85). (Table 2).

## Discussion

In this study of 344 Uganda women who initially tested HIV-negative during pregnancy in 2022, only 18.6% reported receiving retesting prior to delivery. The Uganda guidelines call for HIV testing of all women attending ANC, and those who are negative be retested at all subsequent ANC visits [11]. The findings of this study are concerning as they are far below the national recommendations. In contrast to our findings, a higher HIV retesting rate was reported in Mozambique at 40.7% [27]. Several factors contribute to the low proportion of HIV retesting during ANC in the current study, primarily due to systemic challenges such as inadequate efforts by healthcare providers to educate women on the importance of retesting, the persistent stigma and fear surrounding HIV, and significant barriers within the healthcare system that limit access to testing services [28,29]. This low rate of HIV retesting raises concerns about missed opportunities for timely detection of new HIV infections and subsequent interventions that can prevent vertical transmission [30].

Although studies focusing specifically on HIV retesting among women attending ANC are limited, related studies on HIV testing during ANC have reported varying coverage of HIV retesting during pregnancy. For instance, the proportion of women who retested for HIV during pregnancy in Kenya was 32% [19], while in Sudan it was at 3.6% [31]. Our findings collectively highlight the urgent need for targeted interventions aimed at improving retesting rates, raising awareness, addressing barriers, and strengthening ANC programs in high HIV-burden settings. Some studies have shown that interventions such as ensuring a constant supply of testing equipment, comprehensive health education and counselling for pregnant women, provider training to ensure quality care, and addressing the substantial loss to follow-up of pregnant women during ANC could help in improving HIV retesting [32–34]. Such interventions are crucial to ensure comprehensive HIV care for pregnant women, including increased retesting rates and timely interventions when new HIV infections are identified.

The study found that the number of ANC visits was significantly associated with maternal HIV retesting rates. Pregnant women who attended at least four ANC visits had a higher prevalence of HIV retesting compared to those who had attended less than four visits. Regular and consistent engagement with ANC services provides more opportunities for healthcare providers to offer and promote retesting, increasing the likelihood of pregnant women undergoing HIV retesting. Studies in Brazil and Mozambique have also found an association between the high number of ANC visits and HIV retesting [13,35]. Also, participants who attended ANC in the second trimester had a lower prevalence of HIV retesting compared to those in the first trimester. This suggests that early initiation of ANC plays a crucial role in improving HIV retesting rates. This finding is consistent with findings from studies in Kenya and the United States of America, which found that delayed ANC visits resulted in low HIV retesting [32,36].

It was also found that women whose pregnancy was planned had a higher prevalence of HIV retesting compared to those with unplanned pregnancies. Women who actively plan their pregnancies may have higher awareness and better access to and engagement with healthcare services, including retesting for HIV during ANC [37]. Similarly, a study in South Africa found that women were significantly more likely to present earlier for HIV testing if the pregnancy was planned [38]. Efforts to increase awareness and encourage pregnancy planning are likely to increase maternal HIV retesting. Additionally, mothers who were satisfied with the quality of the healthcare services given at the health facilities were more likely to be retested for HIV. This could be attributed to a positive healthcare experience characterized by good quality care and respectful treatment that motivates women to embrace HIV retesting during ANC. The study findings align with a study conducted in eastern Uganda, which demonstrated that pregnant women who were satisfied with the services were more likely to test for HIV during ANC [39]. Additionally, studies in Brazil and Tanzania reported that good quality services during ANC, such as high-quality HIV counselling and testing services during the initial ANC visit, were associated with increased odds of HIV retesting [40,41]. This finding calls for improving the quality of services offered at health facilities to encourage more HIV retesting among pregnant women.

We also found that the availability of HIV testing resources at the ANC clinic played a crucial role in HIV retesting during ANC. Women who reported having access to testing resources demonstrated higher rates of HIV retesting. This finding emphasizes the importance of well-equipped ANC clinics that provide readily available testing resources, including HIV testing kits, which facilitate the process of HIV retesting. Similar observations have been reported in studies conducted among diverse populations, further emphasizing the significance of resource availability and accessibility in promoting the utilization of retesting services [29,37,42]. Targeted policies and interventions are essential to ensure that ANC clinics are well-equipped with the necessary HIV testing resources. In addition, the implementation of efficient inventory management systems can help monitor and regulate the supply of testing resources, preventing stock shortages and ensuring a continuous supply of essential supplies. Furthermore, integrating technological advancements such as point-of-care testing technologies can also streamline the testing process and improve the accessibility and efficiency of HIV retesting during ANC visits.

### Strength and limitations

This study interviewed women attending ANC rather than relying on facility records which may have been incomplete. However, some limitations were noted; some individual factors reported were assessed based on self-report, and this could have introduced social desirability bias. However, to minimize this, the interviewers were well-trained to probe and clarify responses during interviews. More to this, assurance was given to respondents that the information was only for research purposes and that they were reassured of confidentiality. The study was also only conducted in selected high-level facilities in a rural district; thus, the findings are not generalizable across the district and the country. Nevertheless, the study provides important insights into maternal HIV retesting and can inform the provision and uptake of the intervention. Additionally, we used ANC registers to obtain the data on HIV retesting and did not consider potential retesting from other facilities, which could have underestimated HIV retesting in the area. However, the health facilities that were considered for this study were major health facilities in the study area from which most pregnant women obtain their services. We did not consider the average knowledge and neutral perceptions in our categorization of these two variables, which may have limited the depth of analysis. Future research should consider including these categories to provide more detailed insights and capture nuanced variations in responses.

### Conclusion and implications

The retesting rate for HIV during ANC in health facilities of Mayuge district was found to be low despite national guidelines recommending universal HIV retesting for all HIV-negative women attending ANC. The study found that ANC-related factors at individual and system levels influence maternal HIV retesting. For example, later ANC start, attending fewer than four ANC visits, and having a planned pregnancy. On the other hand, the health system factors that were found to influence ANC retesting were women’s satisfaction with the quality of health services and easy accessibility to HIV testing resources. The study findings underscore the need to address the low retesting rates during ANC for effective prevention of vertical transmission of HIV, for example, through strategies focused on encouraging early and repeated ANC visits, encouraging pregnancy planning through integrating family planning services into ANC care and providing education on contraceptive options. Efforts to the availability of testing resources and enhancing patient satisfaction with care are also key.

## Supporting information

S1 Text Data collection questionnaire. (DOCX)

S1 Data Dataset. (XLSX)
