# Supplementary material for: Maternal HIV retesting during antenatal care in selected health facilities in Mayuge district, Uganda: A cross-sectional study
Source: PLOS Glob Public Health. 2025 Jan 15;5(1):e0004173. doi: 10.1371/journal.pgph.0004173 (PMC11734972; doi:10.1371/journal.pgph.0004173)
Supplement: S1 Text — (DOCX) [file pgph.0004173.s001.docx]

**Study Title**: Prevalence and factors associated with maternal HIV retesting during antenatal care in Mayuge district, Uganda

| **#** | **QUESTIONS** | **RESPONSE** | |
| --- | --- | --- | --- |
|  | **General information** |  | |
|  | Participant ID |  | |
|  | Date of survey |  | |
|  | Time at start of survey |  | |
|  | Healthcare facility name |  | |
| **SOCIO-DEMOGRAPHIC CHARACTERISTICS** | | | |
|  | How old are you? |  | |
|  | Residence | 🞎 Rural  🞎 Urban | |
|  | What is your marital status? | 🞎 Single/never married  🞎 Married/Cohabiting  🞎 Divorced/Separated  🞎 Widowed | |
|  | What is your highest level of education? | 🞎 No formal education  🞎 Primary  🞎 Secondary  🞎 Tertiary | |
|  | What is your main source of income? | 🞎 Unemployed  🞎 Self-employed (Casual laborer, peasant famer, petty trade)  🞎 Employed (by someone or an organization) | |
|  | Does your household have the following items?  *Tick any that apply* | 🞎 Electricity  🞎 Working radio  🞎 Working television  🞎 Working phone (landline or mobile)  🞎 Working refrigerator  🞎 Working solar panel  🞎 Working gas stove | |
|  | Does your household have the following animals?  *Tick any that apply* | 🞎 Chickens, ducks, or other birds.  🞎 Cows, goats, sheep, pigs, camels or other large mammals  🞎 Rabbits, guinea pigs, or other small mammals  🞎 Does not know | |
| **ANC RELATED CHARACTERISTICS** | | | |
|  | Parity | 🞎 Uniparous  🞎 Multiparous | |
|  | Start of antenatal care | 🞎 First trimester (From Conception to 12 weeks  🞎 Second trimester (From 13weeks till 24weeks)  🞎 Third trimester (above 24 week) | |
|  | Number of antenatal visits done |  | |
|  | Was the pregnancy planned? | 🞎 Yes  🞎 No | |
|  | **HIV RETEST UPTAKE DURING ANC** | | |
|  | Weeks of gestation (weeks) at the time of the first HIV test |  | |
|  | When was the first ANC Visit? |  | |
|  | Results of the HIV test at the first ANC visit | 🞎 Negative  🞎 Positive | |
|  | Date when the first HIV test was done | DD/MM/YY | |
|  | Has the next HIV test been done at the second ANC visit? | 🞎 Yes  🞎 No | |
|  | Date when the second ANC visit was done | DD/MM/YY | |
|  | Results of the HIV re-test result at the second ANC visit. | 🞎 Negative  🞎 Positive | |
|  |  |  | |
|  | **KNOWLEDGE OF MTCT** |  | |
| \| **#** \| **Items** \| **Response** \| **Code** \| **Skip** \| \| --- \| --- \| --- \| --- \| --- \| \|  \| What is the mode of transmission of HIV? \| Sexual intercourse \| 1 \|  \| \| Blood and blood product \| 2 \|  \| \| MTCT \| 3 \|  \| \| Injection and blades \| 4 \|  \| \| I don’t know \| 5 \|  \| \|  \| How can one prevent being infected with HIV? \| Abstinence \| 1 \|  \| \| Faithful \| 2 \|  \| \| Use condom \| 3 \|  \| \| I don’t know \| 4 \|  \| \|  \| Have you ever heard of mother to child transmission? \| Yes \| 1 \|  \| \| No \| 2 \|  \| \|  \| Does HIV testing have advantages for PMTCT? \| Yes \| 1 \|  \| \| No \| 2 \|  \| \|  \| How can transmission of HIV from mother to child transmission occur? \| During pregnancy \| 1 \|  \| \| During delivery \| 2 \|  \| \| During breast feeding \| 3 \|  \| \| Others specify \|  \|  \| \|  \| Can mother to child transmission of HIV be prevented? \| Yes \| 1 \|  \| \| No \| 2 \|  \| \|  \| How can we prevent mother to child  transmission of HIV? \| Chemotherapy (ARV) \| 1 \|  \| \|  \| Vaccination \| 2 \|  \| \| Traditional therapy \| 3 \|  \| \| Others (specify) \|  \|  \| | | | |
|  | **INDIVIDUAL PERCEPTIONS** |  | |
|  | Have you ever felt that you needed to get an HIV test? | | Yes  No |
|  | On a scale of 1-5, how concerned are you about the possibility of having contracted HIV since your last test? | | Very concerned  Concerned  Not that concerned  Not at all concerned |
|  | **Perception** | | |
| \| **Statement** \| **Strongly disagree** \| **Disagree** \| **Neutral** \| **Agree** \| **Strongly**  **Agree** \| \| --- \| --- \| --- \| --- \| --- \| --- \| \| I think am at risk of HIV \|  \|  \|  \|  \|  \| \| I think my partner is at risk of HIV \|  \|  \|  \|  \|  \| \| I am worried about getting HIV/AIDS \|  \|  \|  \|  \|  \| \| I will be very sick if I get HIV \|  \|  \|  \|  \|  \| \| If I get HIV, I might require hospitalization \|  \|  \|  \|  \|  \| \| If I get HIV, I might die \|  \|  \|  \|  \|  \| \| Routine HIV testing during antenatal care visits can help prevent mother-to-child transmission of HIV \|  \|  \|  \|  \|  \| \| I don’t have time to get tested \|  \|  \|  \|  \|  \| \| HIV testing takes too long \|  \|  \|  \|  \|  \| \| I am afraid of the HIV test results \|  \|  \|  \|  \|  \| \| I trust the HIV test counselors and nurses to keep my information confidential \|  \|  \|  \|  \|  \| \| It would be embarrassing to get tested for HIV \|  \|  \|  \|  \|  \| \| HIV tests give accurate results \|  \|  \|  \|  \|  \| \| The distance to the healthcare facility is too long for me \|  \|  \|  \|  \|  \| | | | |
|  | **HEALTHCARE SYSTEM FACTORS** | | |
| **Client satisfaction with ANC services**  For each of the following questions, please rate your level of satisfaction with ANC services at the healthcare facility   \| **Question** \| **Very dissatisfied** \| **Dissatisfied** \| **Satisfied** \| **Very satisfied** \| \| --- \| --- \| --- \| --- \| --- \| \| How satisfied are you with the quality of care you have received in this facility? \|  \|  \|  \|  \| \| How satisfied are you with the waiting time? \|  \|  \|  \|  \| \| How satisfied are you with the cleanliness? \|  \|  \|  \|  \| \| How satisfied are you with examination room privacy? \|  \|  \|  \|  \| \| How satisfied are you with consultation room privacy? \|  \|  \|  \|  \| \| How satisfied are you with the time given by health worker? \|  \|  \|  \|  \| \| How satisfied are you with the explanation given by health worker? \|  \|  \|  \|  \| \| How satisfied are you with the availability of medicines? \|  \|  \|  \|  \| \| How satisfied are you with the cost of service? \|  \|  \|  \|  \| \| How satisfied are you with the provider’s attitude? \|  \|  \|  \|  \| \| In an overall sense, how satisfied are you with the antenatal care service you have received? \|  \|  \|  \|  \| | | | |
|  | What is the distance to the nearest Health facility? | <5km  ≥5km | |
|  | How much time does it take you to reach the nearest health facility? | Less than 15 minutes  15-30 minutes  >1 hour | |
|  | The last time you sought care, how long did you wait before seeing a health worker? |  | |
|  | Total time spent at ANC |  | |
|  | While at the healthcare facility, are you given information on HIV testing? *Fliers, sensitization,* | Yes  No | |
|  | Have you been offered HIV testing at the ANC clinic during your antenatal care visits? | | Yes  No  *If yes, answer the next questions* |
|  | Were there any instances when you wanted to get tested for HIV at the ANC clinic, but the testing resources were not available? | | Yes  No |
|  | Were there any delays in getting your HIV test results from the ANC clinic? | | Yes  No |
|  | How long did you have to wait to receive your HIV test results? | | Within a day  Within a week  Within two weeks  Longer than two weeks |
|  | Were the HIV testing resources at the ANC clinic easily accessible? | | Yes  No |
|  | Did you receive pre-test counseling before getting an HIV test at the ANC clinic? | | Yes  No |
|  | During your antenatal care visits at the ANC clinic, were there enough healthcare providers available to provide quality care, including HIV testing? | | Yes, always  Sometimes  Rarely  No, never |
|  | **SOCIAL SUPPORT** |  | |
|  | ***Spousal support and strain***  For each of the following questions, please rate your partner’s support on a 4-point Likert scale   \| **Question** \| **A lot** \| **Somehow** \| **A little** \| **Not at all** \| \| --- \| --- \| --- \| --- \| --- \| \| How much does your spouse or partner really care about you? \|  \|  \|  \|  \| \| How much does he understand the way you feel about things? \|  \|  \|  \|  \| \| How much does he appreciate you? \|  \|  \|  \|  \| \| How much do you rely on him for help if you have a serious problem? \|  \|  \|  \|  \| \| How much can you open up to him if you need to talk about your worries? \|  \|  \|  \|  \| \| How much can you relax and be yourself around him? \|  \|  \|  \|  \|  \| **Question** \| **Often** \| **Sometimes** \| **Rarely** \| **Never** \| \| --- \| --- \| --- \| --- \| --- \| \| How often does your spouse make too many demands on you? \|  \|  \|  \|  \| \| How often does he criticize you? \|  \|  \|  \|  \| \| How often does he let you down when you are counting on him? \|  \|  \|  \|  \| \| How often does he get on your nerves? \|  \|  \|  \|  \| | | |
|  | ***Family support and strain***   \| **Question** \| **A lot** \| **Somehow** \| **A little** \| **Not at all** \| \| --- \| --- \| --- \| --- \| --- \| \| Not including your spouse or partner, how much do members of your family really care about you? \|  \|  \|  \|  \| \| How much do they understand the way you feel about things? \|  \|  \|  \|  \| \| How much do they appreciate you? \|  \|  \|  \|  \| \| How much can you rely on them for help if you have a serious problem? \|  \|  \|  \|  \| \| How much can you open up to them if you need to talk about your worries? \|  \|  \|  \|  \| \| How much can you relax and be yourself around him? \|  \|  \|  \|  \|  \| **Question** \| **Often** \| **Sometimes** \| **Rarely** \| **Never** \| \| --- \| --- \| --- \| --- \| --- \| \| Not including your spouse or partner, how often do members of your family make too many demands on you? \|  \|  \|  \|  \| \| How often do they criticize you? \|  \|  \|  \|  \| \| How often do they let you down when you are counting on them? \|  \|  \|  \|  \| \| How often do they get on your nerves? \|  \|  \|  \|  \| | | |
